# Association of serum phosphate and changes in serum phosphate with 28-day mortality in septic shock from MIMIC-IV database

**DOI:** 10.1038/s41598-023-49170-6

**Published:** 2023-12-10

**Authors:** Wenbin Nan, Qiong Huang, Jinfa Wan, Zhenyu Peng

**Affiliations:** 1 Department of Emergency Medicine, Second Xiangya Hospital, Central South University, Changsha, 410011 People’s Republic of China; 2 Emergency Medicine and Difficult Diseases Institute, Central South University, Changsha, 410011 People’s Republic of China; 3 Department of Geriatric Respiratory and Critical Care Medicine, Xiangya Hospital, Central South University, Changsha, 410008 People’s Republic of China; 4 Department of Geriatric Medicine, Xiangya Hospital, Central South University, Changsha, 410008 People’s Republic of China; 5 National Clinical Research Center for Geriatric Disorders, Xiangya Hospital, Central South University, Changsha, 410008 People’s Republic of China

**Keywords:** Biomarkers, Cardiology, Risk factors

## Abstract

This study aimed to investigate the relationship between serum phosphate levels, changes in serum phosphate levels, and 28-day mortality in patients with septic shock. In this retrospective study, data were collected from the Medical Information Mart for Intensive Care IV (MIMIC-IV) database between 2008 and 2019. Patients were divided into three groups according to the tertiles of serum phosphate levels. Kaplan–Meier curves and log-rank test analyses were used for survival analysis. Multivariate logistic regression, and restricted cubic spline (RCS) curve were used to explore the association between serum phosphate, delta serum phosphate levels and 28-day mortality. In total, 3296 patients with septic shock were included in the study, and the 28-day mortality was 30.0%. Serum phosphate levels were significantly higher in the non-survivor group than in the survivor group. The Kaplan–Meier curves showed significant differences among the three groups. Multivariate logistic regression analysis and the RCS curve showed that serum phosphate levels were independently and positively associated with the 28-day mortality of septic shock. Non-survivors had higher delta serum phosphate levels than survivors. Survival analysis showed that patients with higher delta serum phosphate levels had higher 28-day mortality. A non-linear relationship was detected between delta serum phosphate and 28-day mortality with a point of inflection at − 0.3 mg/dL. Serum phosphate levels were positively and independently associated with 28-day mortality in septic shock. Delta serum phosphate level was a high-risk factor for patients with septic shock.

## Introduction

Sepsis is a life-threatening organ dysfunction caused by a non-homeostatic host response to infection. Septic shock is a lethal complication of sepsis characterized by persistent tissue hypoperfusion after adequate fluid resuscitation^1^. Numerous studies have reported that septic shock affects 10%–30% of patients admitted to the intensive care unit (ICU) and causes an increased mortality of approximately 45–63%^2–4^. Despite the adoption of multiple measures, septic shock is still associated with high mortality, prolonged hospitalization, and increased hospital costs, and has become a major public health issue worldwide^2,5,6^. Early identification of high-risk factors in patients with septic shock might help guide clinical practice and reduce mortality rates^7^.

Phosphorus plays a crucial role in the maintenance of cellular integrity and organ function^8–10^. Phosphate refers to the inorganic phosphorus that exerts multiple physiological functions, such as membrane transport, energy metabolism, skeletal mineralization, and muscle contraction^9,11^. Numerous studies have revealed that higher serum phosphate levels are associated with adverse outcomes in various diseases, including chronic kidney disease (CKD), acute ischemic stroke, blunt trauma, and chronic obstructive pulmonary disease (COPD)^11–14^. Several recent studies have indicated that serum phosphate disturbances contribute to worse sepsis outcomes^8,11,15,16^. However, the association between serum phosphate levels and 28-day mortality in patients with septic shock remains unclear. Therefore, in this retrospective study, we aimed to explore the relationship between serum phosphate levels and 28-day mortality in patients with septic shock using the Medical Information Mart for Intensive Care IV (MIMIC-IV) database.

## Materials and methods

### Data source

All the data analyzed in our study were extracted from the MIMIC-IV database. The MIMIC-IV database is a large and publicly accessible critical care database that consists of more than 60,000 patients admitted to the Beth Israel Deaconess Medical Center (BIDMC) between 2008 and 2019. We were permitted to access the database after completing online training in the Collaborative Institutional Training Initiative (CITI) program (Record ID: 46,785,473 for Zhenyu Peng). Our study was performed in accordance with the Strengthening the Reporting of Observational Studies in Epidemiology (STROBE) statement.

### Study population

**Figure 1:**
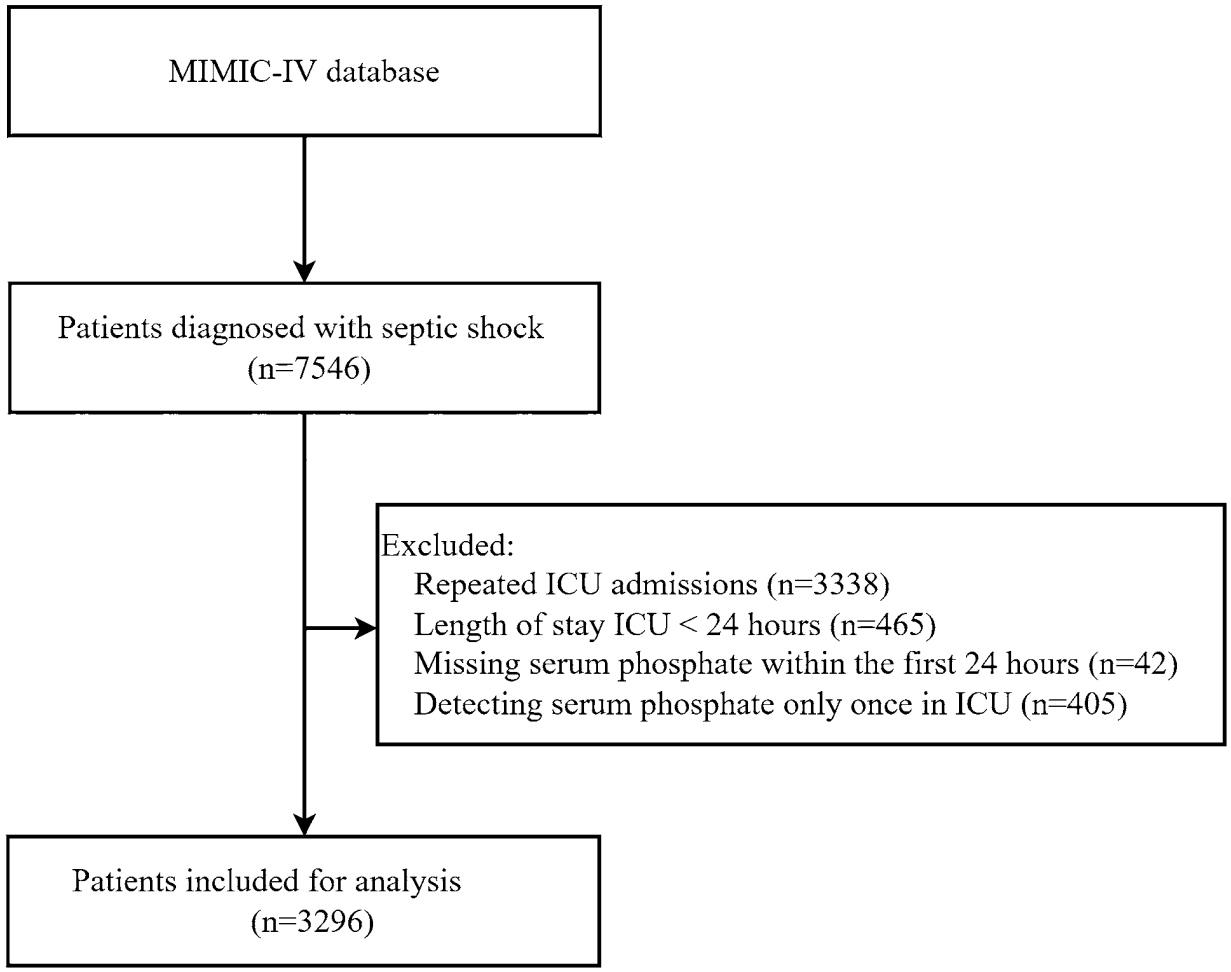
Flow chart of patient’s enrollment. MIMIC-IV—Medical Information Mart for Intensive Care IV; ICU—intensive care unit.

Adult patients diagnosed with septic shock were enrolled in this study according to the ICD-9 diagnostic code 78,552 and ICD-10 diagnostic code R6521 in the MIMIC-IV database (n = 7546). The following patients were excluded: (1) those with more than one ICU admission (n = 3338); (2) length of ICU stay less than 24 h (n = 465); (3) those without serum phosphate measurement in the first 24 h of ICU admission (n = 42); and (4) those without two or more detections of serum phosphate (n = 405); Finally, as shown in Fig. 1, 3296 patients diagnosed with septic shock were included in this study.

### Data extraction

The following data were extracted by pgAdmin4 PostgreSQL from the MIMIC-IV database: (1) demographic characteristics: age, gender and weight; (2) vital signs: temperature, mean arterial pressure (MAP), saturation of peripheral oxygen (SpO_2_), heart rate, and respiratory rate; (3) laboratory tests: white blood cells (WBC), creatinine, sodium, calcium, serum phosphate, and lactate; (4) infection site: respiratory system, urinary system, digestive system and other sites; (5) comorbidities: congestive heart failure, chronic pulmonary disease, diabetes, renal disease, liver disease, malignant tumor, cerebrovascular disease, and peripheral vascular disease; (6) interventions: renal replacement therapy (RRT), ventilation and vasopressor use; (7) severity score: simplified acute physiology score (SAPS II) and sequential organ failure assessment (SOFA); (8) outcomes: ICU stay, in-hospital stay, ICU mortality, in-hospital mortality and 28-day mortality.

### Statistical analysis

The sample size for our study was determined using conventional parameters—80% statistical power and a 5% significance level. Initially estimated at 133–198 patients based on prior research^17–19^, we increased the sample size to 3296 patients to enhance robustness. Continuous variables that were normally distributed or skewed were expressed as mean ± standard deviation (SD) or median with the first and third quartiles, respectively. Categorical variables were presented as numbers and percentages. Statistical differences were analyzed using Student’s *t*-test, Kruskal Wallis H test, Chi-squared test, or one way ANOVA, as appropriate. Patients with septic shock were divided into three groups based on the tertiles of serum phosphate values: T1 group (serum phosphate < 3.2 mg/dL, n = 1115), T2 group (3.2 mg/dL ≤ serum phosphate < 4.5 mg/dL, n = 1108) and T3 group (serum phosphate ≥ 4.5 mg/dL, n = 1073). Univariate and multivariate logistic regression analyses were performed to evaluate the hazard ratio (HR) of the covariates for 28-day mortality. We constructed Kaplan–Meier curves to illustrate the survival of patients in the different groups. Three models were used to minimize the effects of confounding factors. The crude model was not adjusted for the covariates. Model I was adjusted for age, gender, and weight. Model II was adjusted for all covariates in this study. A restricted cubic spline (RCS) curve was performed to reveal the dose–response relationship between serum phosphate levels and 28-day mortality. Moreover, the delta serum phosphate level was calculated as the difference between the initial serum phosphate level and the last serum phosphate level measured in the ICU. Kaplan–Meier curves were used to assess the 28-day survival probabilities of the high and low-delta serum phosphate groups. We performed RCS curve to determine the association between delta serum phosphate level and 28-day mortality. Stata version 15.0 (College Station, Texas, USA) and R software version 4.2.0 (R Foundation, Vienna, Austria) were used to perform the statistical analyses in this study. Statistical significance was defined as a two-sided *P* < 0.05.

### Institutional review board statement

The MIMIC-IV database was approved by the Massachusetts Institute of Technology (Cambridge, MA) and Beth Israel Deaconess Medical Center (Boston, MA), and informed consent was obtained for the original data collection. All procedures performed in studies involving human participants were in accordance with the ethical standards of the institutional and national research committee and with the 1964 Helsinki declaration and its later amendments or comparable ethical standards.

## Results

### Baseline characteristics

As shown in Supplementary Table 1 and Supplementary Table 2, 3296 eligible patients with septic shock were enrolled in the study. Of these patients, 989 (30.0%) died within 28 days of ICU admission. Serum phosphate levels were significantly higher in the non-survivor group than in the survivor group. All patients were categorized into the T1 (n = 1115), T2 (n = 1108) and T3 (n = 1073) groups according to the tertiles of serum phosphate values. As displayed in Table 1, the patients in the T3 group had a higher proportion of males, and higher weight, WBC, creatinine, lactate, SAPS II, and SOFA as well as a higher prevalence of congestive heart failure, diabetes, renal disease, liver disease, and peripheral vascular disease. However, temperature, MAP, and sodium levels were lower in the T3 group. Interventions such as ventilation, RRT, and vasopressor use were frequently required in the T3 group. Patients with higher serum phosphate levels had longer ICU stay, and higher ICU mortality, in-hospital mortality, and 28-day mortality.

**Table 1:** Baseline characteristics of the patients.

	T1 group (n = 1115)	T2 group (n = 1108)	T3 group (n = 1073)	*P* value
Age (years)	66.95 (55.68, 79.85)	70.8 (59.17, 81.24)	68.12 (56.83, 78.47)	< 0.001
Gender, n (%)				
Female	556 (49.87)	502 (45.31)	429 (39.98)	< 0.001
Male	559 (50.13)	606 (54.69)	644 (60.02)	
Weight (kg)	76.7 (64.4, 91.6)	77.5 (64, 95.89)	85.7 (69, 102.2)	< 0.001
Temperature (℃)	37.04 (36.72, 37.5)	36.87 (36.61, 37.23)	36.81 (36.52, 37.19)	< 0.001
MAP (mm Hg)	74.77 (70.71, 79.68)	74.26 (69.99, 79.23)	73.74 (69.48, 79.05)	< 0.001
SpO_2_ (%)	96.74 (95.32, 98.09)	96.91 (95.43, 98.27)	96.75 (95.3, 98.2)	0.188
Heart rate (bpm)	92.18 (80.64, 106.14)	92.69 (79.36, 104.52)	91.73 (80.06, 104.74)	0.572
Respiratory rate (bpm)	21.2 (18.23, 24.21)	20.67 (17.93, 24)	21.36 (18.46, 24.84)	0.004
WBC (10^9^/L)	12.4 (7.65, 18.55)	14.4 (9.2, 20.6)	15.25 (10, 21.77)	< 0.001
Creatinine (mg/dL)	1.0 (0.7, 1.4)	1.3 (0.9, 2)	2.4 (1.6, 4)	< 0.001
Sodium (mmol/L)	139 (135, 142)	138 (135, 141)	137 (133, 141)	< 0.001
Calcium (mg/dL)	7.7 (7.1, 8.2)	7.9 (7.3, 8.4)	7.9 (7.3, 8.6)	< 0.001
Lactate (mmol/L)	2.0 (1.42, 3)	2.1 (1.4, 3.2)	2.5 (1.6, 4.6)	< 0.001
Infection site, n (%)				
Respiratory system	429 (38.48)	425 (38.36)	414 (38.58)	0.994
Urinary system	306 (27.44)	293 (26.44)	256 (23.86)	0.144
Digestive system	263 (23.59)	277 (25)	256 (23.86)	0.712
Other sites	317 (28.43)	326 (29.42)	339 (31.59)	0.256
Congestive heart failure, n (%)	291 (26.1)	397 (35.83)	405 (37.74)	< 0.001
Chronic pulmonary disease, n (%)	271 (24.3)	340 (30.69)	298 (27.77)	0.003
Diabetes, n (%)	322 (28.88)	360 (32.49)	370 (34.48)	0.017
Renal disease, n (%)	167 (14.98)	266 (24.01)	387 (36.07)	< 0.001
Liver disease, n (%)	222 (19.91)	243 (21.93)	346 (32.25)	< 0.001
Malignant tumor, n (%)	217 (19.46)	248 (22.38)	206 (19.2)	0.120
Cerebrovascular disease, n (%)	117 (10.49)	119 (10.74)	102 (9.51)	0.605
Peripheral vascular disease, n (%)	97 (8.7)	112 (10.11)	142 (13.23)	0.002
RRT, n (%)	82 (7.35)	194 (17.51)	395 (36.81)	< 0.001
Ventilation, n (%)	607 (54.44)	714 (64.44)	819 (76.33)	< 0.001
Vasopressor use, n (%)	913 (81.88)	951 (85.83)	962 (89.66)	< 0.001
SAPSII	42 (33, 51)	46 (37, 55)	53 (44, 64)	< 0.001
SOFA	8 (6, 11)	10 (7, 12)	12 (9, 15)	< 0.001
ICU stay	3.99 (2.46, 8.07)	4.74 (2.82, 9.57)	5.72 (2.78, 10.63)	< 0.001
In-hospital stay	10.9 (6.46, 19.87)	12.68 (6.89, 21.35)	12.43 (6.26, 22.65)	0.030
ICU mortality, n (%)	162 (14.53)	268 (24.19)	368 (34.3)	< 0.001
In-hospital mortality, n (%)	269 (24.13)	362 (32.67)	483 (45.01)	< 0.001
28-day mortality, n (%)	230 (20.63)	317 (28.61)	442 (41.19)	< 0.001

*MAP* mean arterial pressure; *SpO*_*2*_ saturation of peripheral oxygen; *WBC* white blood cell; *RRT* renal replacement therapy; *SAPS II* simplified acute physiology score II; *SOFA* sequential organ failure assessment; *ICU* intensive care unit.

### Univariate and multivariate analyses

Univariate and multivariate analyses were performed to assess the HR of the covariates for 28-day mortality in patients with septic shock as shown in the Supplementary Table 3. Univariate analysis showed that age, temperature, MAP, SpO_2_, heart rate, respiratory rate, creatinine, lactate, urinary system infection, digestive system infection, congestive heart failure, renal disease, liver disease, malignant tumor, peripheral vascular disease, RRT, ventilation, vasopressor use, SAPSII, SOFA and serum phosphate levels were associated with 28-day mortality. Multivariate analysis demonstrated that age, temperature, MAP, SpO_2_, heart rate, respiratory rate, creatinine, calcium, urinary system infection, digestive system infection, liver disease, malignant tumor, ventilation, SAPS II, SOFA, and serum phosphate levels were associated with 28-day mortality after adjusting for confounding factors.

### Association between serum phosphate and 28-day mortality

**Figure 2:**
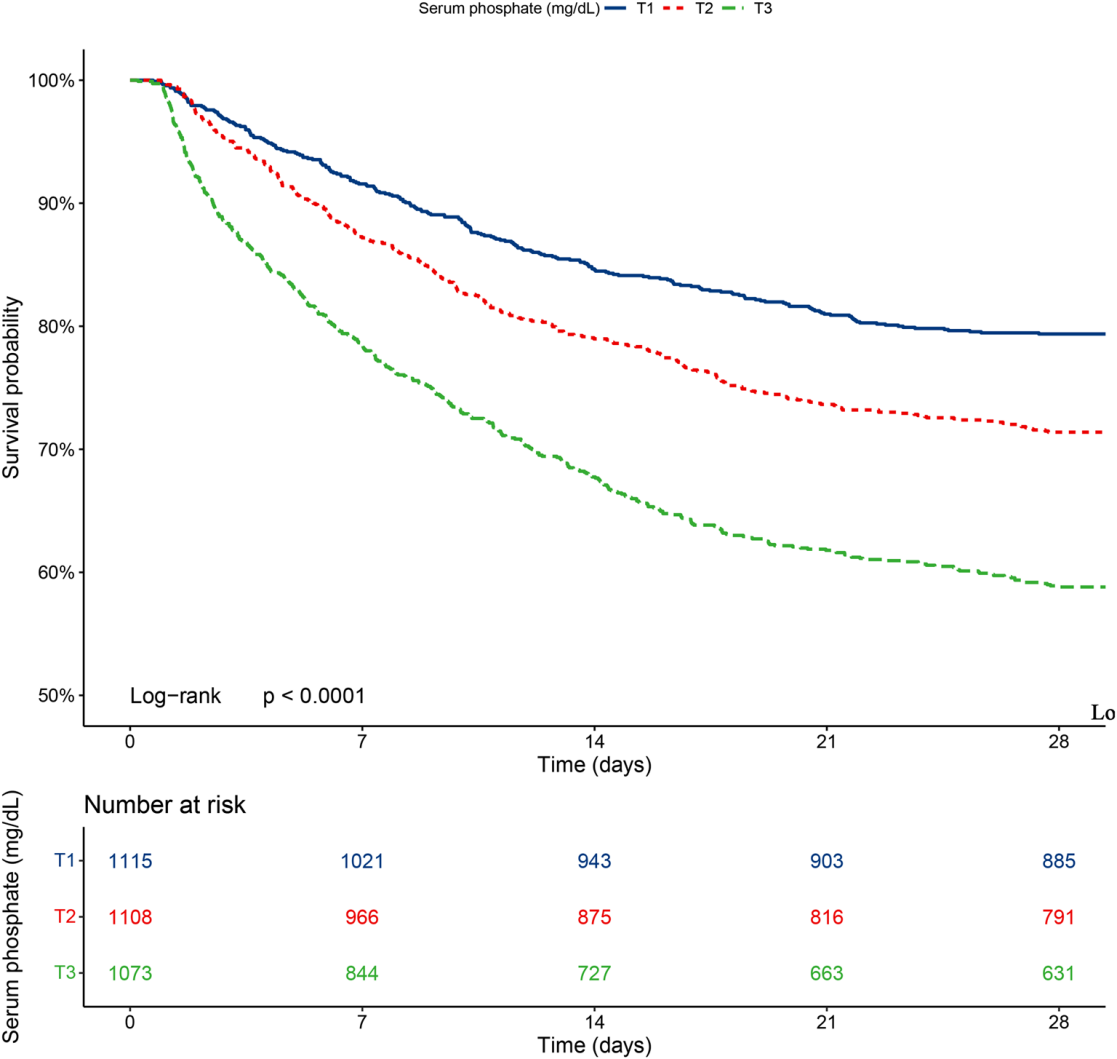
Kaplan–Meier survival analysis plots for 28-day mortality with serum phosphate category. Blue line, T1 group; Red line, T2 group; Green line, T3 group.

**Figure 3:**
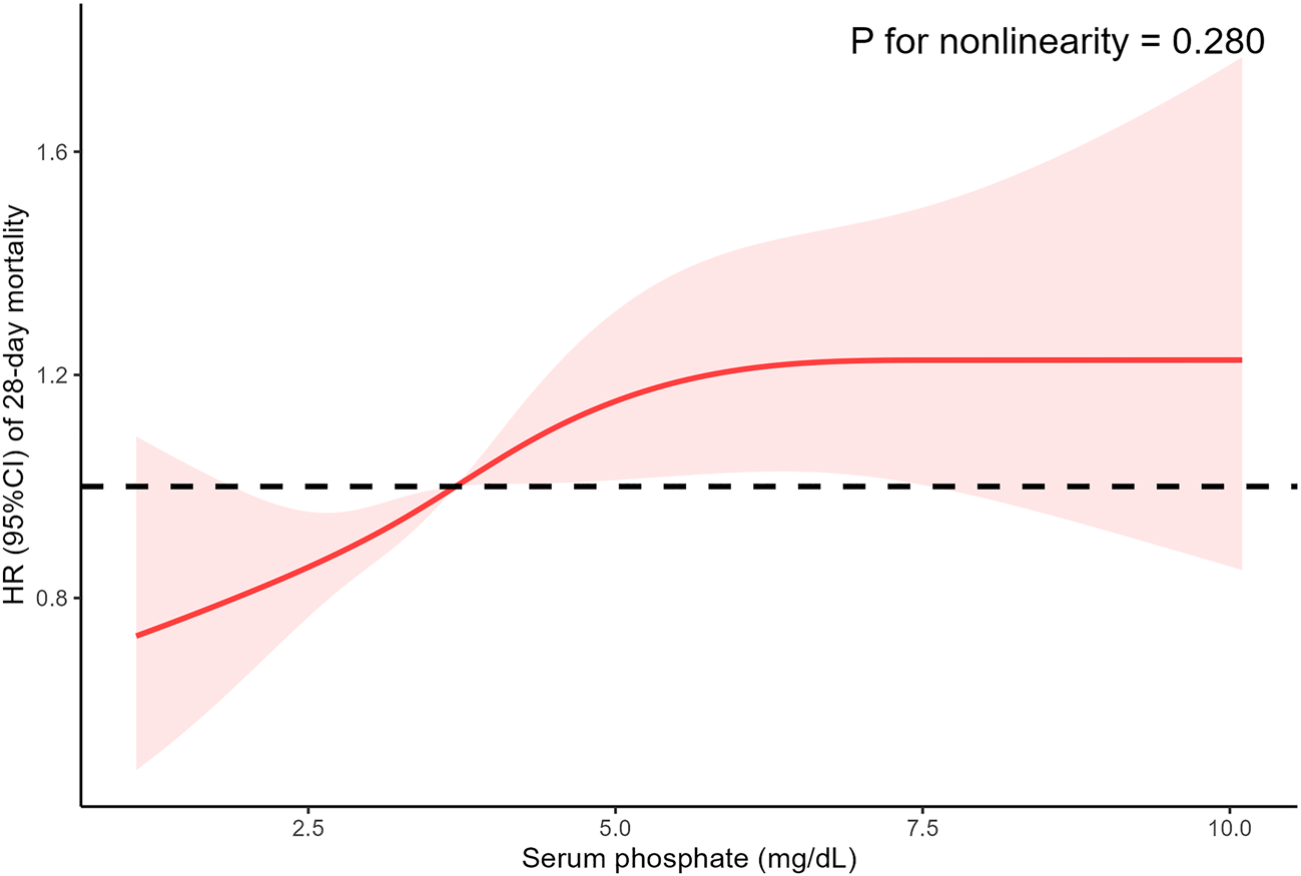
Association between serum phosphate levels and 28-day mortality using a RCS curve. The solid line and shadow represented the HR of 28-day mortality and 95% CI, respectively. The RCS curve was adjusted for all covariates. RCS, restricted cubic spline; HR, hazard ratio; CI, confidence interval.

Kaplan–Meier curves were constructed to illustrate the survival of patients with septic shock in the different groups. As shown in Fig. 2, the 28-day mortality rate was significantly higher in the T3 group than in the T2 and T1 groups (log-rank *p* < 0.001). The crude model, model I, and model II were used to explore the association between serum phosphate levels and 28-day mortality in septic shock. As shown in Table 2, serum phosphate levels were positively correlated with increased risk of 28-day mortality in the crude model (HR = 1.20, 95%CI:1.17–1.24, *P* < 0.001), model I (HR = 1.22, 95%CI:1.18–1.26, *P* < 0.001) and model II (HR = 1.07, 95% CI:1.02–1.12, *P* = 0.003). Serum phosphate levels were converted from continuous variables to categorical variables. Patients in the T3 group demonstrated a higher risk of 28-day mortality than those in the T1 group in all three models (T3 in crude model: HR = 2.34, 95%CI: 2.00–2.75, *P* < 0.001, *P* for trend < 0.001; T3 in model I: HR = 2.35, 95%CI: 2.00–2.75, *P* < 0.001, *P* for trend < 0.001; T3 in model II: HR = 1.40, 95%CI: 1.16–1.69, *P* = 0.001, *P* for trend = 0.001). The RCS curve was used to assess the dose–response relationship between serum phosphate levels and 28-day mortality. As shown in Fig. 3, a linear association was discovered between serum phosphate levels and 28-day mortality after adjusting for all confounders (*P* for non-linearity = 0.2).

**Table 2:** Association between serum phosphate and 28-day mortality.

	Crude model		Model I		Model II	
	Crude HR (95%CI)	*P* value	Adjusted HR (95%CI)	*P* value	Adjusted HR (95%CI)	*P* value
Serum phosphate	1.20 (1.17–1.24)	< 0.001	1.22 (1.18–1.26)	< 0.001	1.07 (1.02–1.12)	0.003
T1 group	Reference		Reference		Reference	
T2 group	1.46 (1.23–1.72)	< 0.001	1.39 (1.17–1.65)	< 0.001	1.15 (0.97–1.38)	0.107
T3 group	2.34 (2.00–2.75)	< 0.001	2.35 (2.00–2.75)	< 0.001	1.40 (1.16–1.69)	0.001
*P* for trend	< 0.001		< 0.001		0.001	

*HR* hazard ratio; *CI* confidence interval.

Crude model was not adjusted for any covariates.

Model I was adjusted for age, gender, weight. Model II was adjusted for all covariates.

### Association between delta serum phosphate and 28-day mortality

As shown in Supplementary Figure 1, non-survivors had higher delta serum phosphate level than survivors. As shown in Supplementary Figure 2, Kaplan–Meier survival analysis demonstrated that patients with high delta serum phosphate level had higher 28-day mortality than those with low delta serum phosphate. Furthermore, as shown in Supplementary Figure 3, the RCS curve demonstrated a non-linear relationship between delta serum phosphate and 28-day mortality after adjusting for all confounders (*P* for non-linearity < 0.01). We used a linear regression model and a two-piecewise linear regression model to explore the association between delta serum phosphate level and 28-day mortality. As shown in Table 3, the two-piecewise linear regression model was superior for fitting the association because the p value for the log-likelihood ratio test was < 0.05. The inflection point of delta serum phosphate was − 0.3 mg/dL by threshold effect analysis. There was a positive association between them at delta serum phosphate level ≤  − 0.3 mg/dL (HR 1.07, 95% CI 1.01–1.04, *P* = 0.0331). The risk of 28-day mortality was increased significantly at delta serum phosphate level >  − 0.3 mg/dL (HR 1.30, 95% CI 1.25–1.36, *P* < 0.0001).

**Table 3:** The results of the two-piecewise linear model.

	HR (95%CI)	*P* value
Fitting model by standard linear regression	1.20 (1.17, 1.24)	< 0.001
Fitting model by two-piecewise linear regression		
Inflection point of delta serum phosphate	− 0.3 (mg/dL)	
≤ − 0.3 (mg/dL)	1.07 (1.01, 1.14)	0.033
> − 0.3 (mg/dL)	1.30 (1.24, 1.36)	< 0.001
P for log-likelihood ratio test	< 0.001	

*HR* hazard ratio; *CI* confidence interval.

The model was adjusted for all covariates.

## Discussion

Septic shock is characterized by profound circulatory, cellular, and metabolic abnormalities and is the leading cause of death in hospitals^1–3,20^. Numerous studies have revealed that abnormalities in serum phosphate levels are associated with worse outcomes in various diseases^21–24^. However, the relationship between serum phosphate levels and 28-day mortality in patients with septic shock remains unclear. In this retrospective study, we analyzed 3296 patients with septic shock from the MIMIC-IV database and found that the non-survival group had significantly higher serum phosphate levels than the survival group. Serum phosphate levels were independently positively associated with and 28-day mortality in patients with septic shock. Delta serum phosphate level was significantly higher in non-survivors of septic shock. Patients with higher serum delta serum phosphate level had worse outcomes in patients with septic shock. The relationship between delta serum phosphate level and 28-day mortality was non-linear with a point of inflection at − 0.3 mg/dL.

In recent years, a series of risk factors have been identified as predictors of death from septic shock, including old age, serum lactate level, red blood cell distribution width, blood urea nitrogen level, creatinine level, and SOFA score^25–30^. Despite the development of therapeutic agents and strategies for treating septic shock, the mortality rate remains consistently high among critically ill patients^4,31–34^. Therefore, there is an urgent need to identify more effective indicators for evaluating outcomes of septic shock. Serum phosphate level is an easily accessible parameter in clinical setting^35–37^. Several studies have demonstrated that abnormal serum phosphate levels contribute to adverse outcomes in patients with various diseases. In the Chronic Renal Insufficiency Standards Implementation Study (CRISIS), Eddington et al. demonstrated that higher phosphate levels were associated with increased mortality in non-dialysis patients with CKD stages 3 and 4^12^. Similarly, Campos-Obando et al. reported that hyperphosphatemia was related to increased all-cause mortality and COPD mortality in men based on the Rotterdam Study^14^. An observational study by Kim et al. showed that hyperphosphatemia was a strong predictor of 30-day mortality in patient with blunt trauma ^11^. Zhong et al. observed a U-shaped association between serum phosphate levels and all-cause mortality in a retrospective cohort study of 2944 patients with acute ischemic stroke^13^. Accumulating evidence has indicated that higher serum phosphate levels are significantly associated with worse outcomes in patients with sepsis^16,38,39^. However, no relevant studies have focused on the relationship between serum phosphate levels and the prognosis of septic shock. In the present study, we found that the serum phosphate level was positively and independently associated with the 28-day mortality of patients with septic shock after adjusting for potential confounders. Therefore, the serum phosphate level is a high-risk factor for death due to septic shock.

Phosphate is dynamically changing in the body^8,15^. Several studies have identified that changes in serum phosphate levels are predictive factors for adverse outcomes in critically ill patients^21–24^. Dekker et al. found that changes in serum phosphate levels during high-flux hemodialysis or hemodiafiltration are strongly related to the calcification propensity in dialysis patients^40^. Kim et al. showed that an increase in phosphate level at 48 h (delta phosphate > 0) was associated with an 8.62-fold increased risk of all-cause mortality in patients with acute kidney injury (AKI) undergoing continuous venovenous hemodiafiltration^41^. Wang et al. reported that delta phosphate level was associated with 28-day mortality in patients with septic AKI in a retrospective cohort^42^. However, the association between changes in serum phosphate levels and the 28-day mortality due to septic shock remains unclear. In this study, we found that higher delta serum phosphate level was associated with a higher risk of 28-day mortality in patients with septic shock. 28-day mortality increased dramatically when delta serum phosphate was ≥ -0.3 mg/dL. Therefore, delta serum phosphate level is a high-risk factor for patients with septic shock.

Hyperphosphatemia commonly occurs in patients with increased catabolism, tissue destruction, crush injuries, rhabdomyolysis, or hyperthermia^15,43^. Recent numerous studies have demonstrated that hyperphosphatemia is observed in various diseases. Manghat et al. reported that systemic infections caused cellular breakdown and release phosphate from the cells into the extracellular fluid, contributing to hyperphosphatemia^44^. Opie et al. demonstrated the increased coronary venous inorganic phosphate concentration caused by ATP utilization in hypoxic cardiomyocytes^45^. Tranquada et al. found that lactic acidosis transferred intracellular phosphate into the circulation, resulting in hyperphosphatemia during shock^46^. Therefore, systemic infections, tissue hypoperfusion, and lactic acidosis might contribute to hyperphosphatemia in septic shock. The mechanisms underlying the relationship between serum phosphate levels and mortality in patients with septic shock have not yet been fully elucidated. Several mechanisms may explain these observations. Hyperphosphatemia causes endothelial dysfunction and vascular calcification, resulting in impaired microcirculatory blood flow and organ dysfunction^39,47^. Accumulating evidences demonstrates that hyperphosphatemia contributes to inflammation, oxidative stress, and mitochondrial dysfunction, all of which are involved in the pathogenesis of septic shock^16,35^. Further studies are required to elucidate these mechanisms.

The strengths of this study are as follows: Firstly, it was a large cohort study with high-quality data from the MIMIC-IV database. Secondly, we adjusted potential confounders and reached representative and reliable conclusions. Thirdly, this is the first study to investigate the relationship between dynamic changes in serum phosphate levels and the 28-day mortality in patients with septic shock. However, this study has several limitations. Firstly, bias could not be avoided due to missing data and unmeasured variables in this retrospective study. Secondly, the diagnosis of septic shock was based on ICD-9 and ICD-10 codes and was different from sepsis 3.0, which might have limited generalizability. Thirdly, although we adjusted for creatinine and renal disease as covariates, it would have been better to exclude patients with renal dysfunction from further studies. Finally, data related to the consumption of foods containing inorganic phosphorus additives were unavailable in the MIMIC IV database. Therefore, multicenter prospective studies are required to confirm our findings.

## Conclusion

Serum phosphate levels were positively and independently associated with 28-day mortality in septic shock. Delta serum phosphate level was a high-risk factor for patients with septic shock.

### Supplementary Information

Supplementary Information 1.

Supplementary Information 2.

Supplementary Information 3.

Supplementary Information 4.

Supplementary Information 5.

Supplementary Information 6.

Supplementary Information 7.

## Data Availability

Publicly available datasets were analyzed in this study. This data can be found on the MIMIC-IV database (https://mimic.physionet.org/).
