# Supplementary figures and images for: Association of serum phosphate and changes in serum phosphate with 28-day mortality in septic shock from MIMIC-IV database
Source: Sci Rep. 2023 Dec 10;13:21869. doi: 10.1038/s41598-023-49170-6 (PMC10711004; doi:10.1038/s41598-023-49170-6)

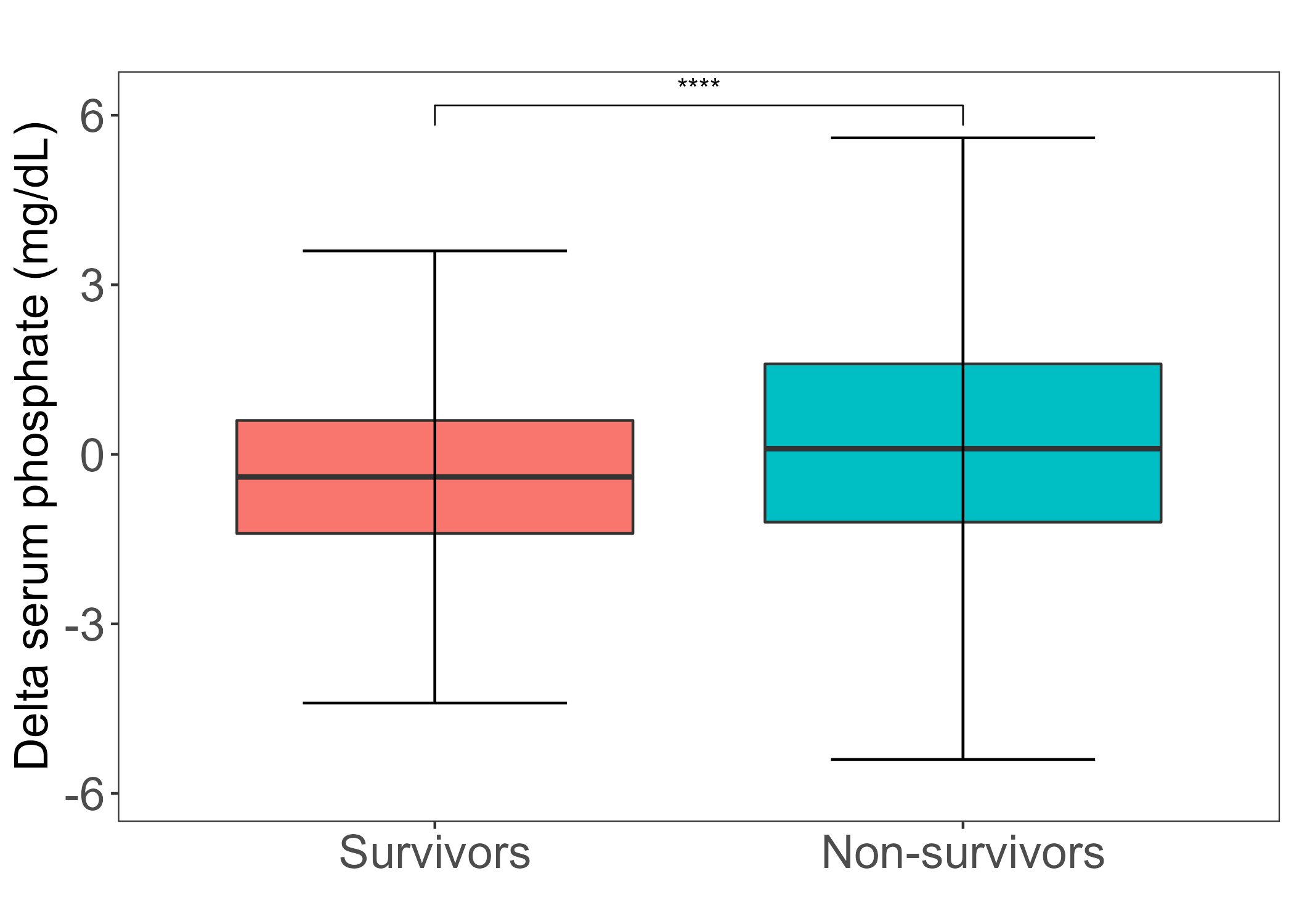

Supplement: Supplementary file 2 — Supplementary Information 2. [file 41598_2023_49170_MOESM2_ESM.tif]

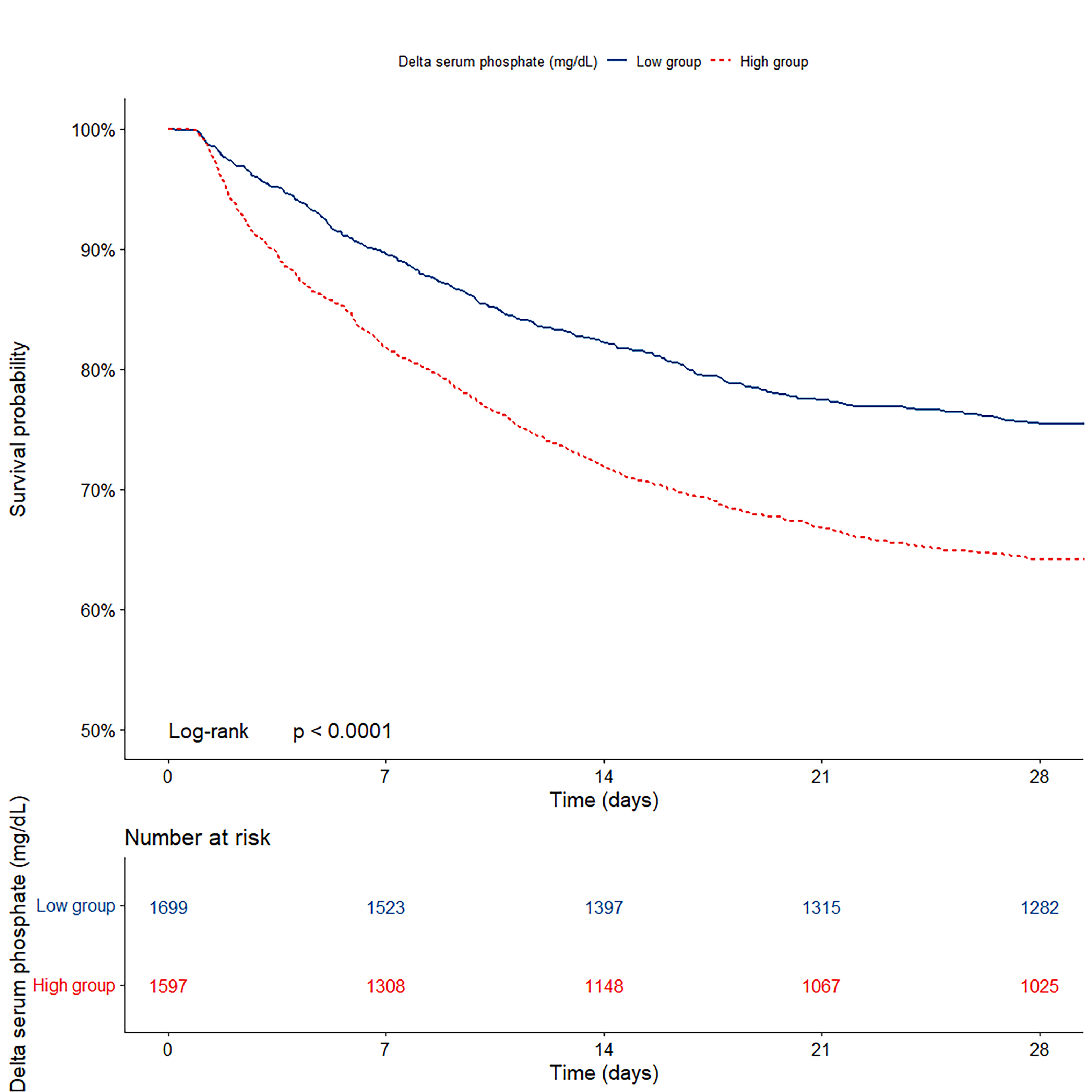

Supplement: Supplementary file 4 — Supplementary Information 4. [file 41598_2023_49170_MOESM4_ESM.tif]

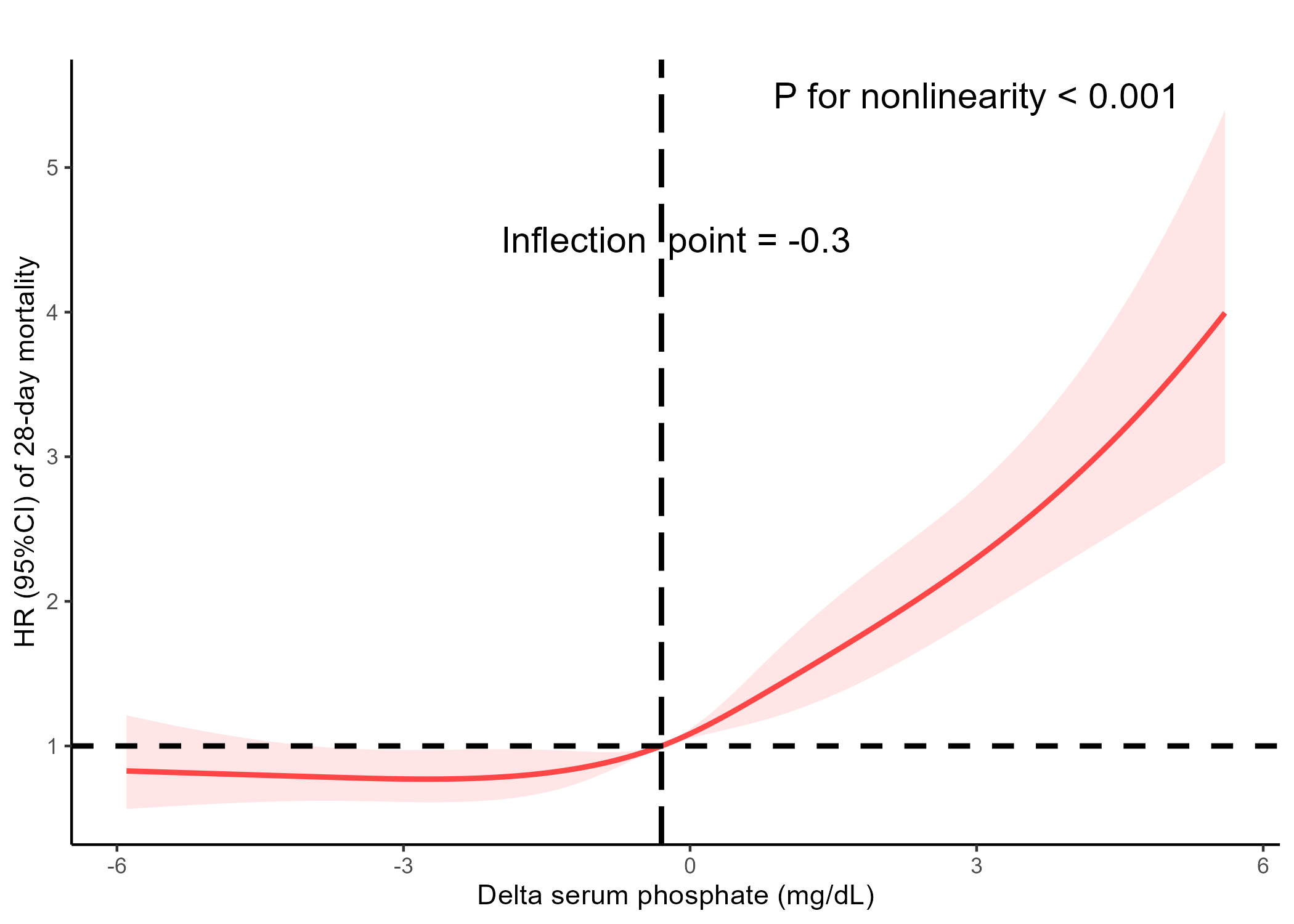

Supplement: Supplementary file 6 — Supplementary Information 6. [file 41598_2023_49170_MOESM6_ESM.tif]
