# Supplementary material for: Association of serum phosphate and changes in serum phosphate with 28-day mortality in septic shock from MIMIC-IV database
Source: Sci Rep. 2023 Dec 10;13:21869. doi: 10.1038/s41598-023-49170-6 (PMC10711004; doi:10.1038/s41598-023-49170-6)
Supplement: Supplementary file 3 — Supplementary Information 3. [file 41598_2023_49170_MOESM3_ESM.docx]

**Supplementary Figure 2.** Kaplan–Meier survival analysis plots for 28-day mortality with delta serum phosphate category. Blue line, low group; Red line, high group.
