# Supplementary material for: Association of serum phosphate and changes in serum phosphate with 28-day mortality in septic shock from MIMIC-IV database
Source: Sci Rep. 2023 Dec 10;13:21869. doi: 10.1038/s41598-023-49170-6 (PMC10711004; doi:10.1038/s41598-023-49170-6)
Supplement: Supplementary file 5 — Supplementary Information 5. [file 41598_2023_49170_MOESM5_ESM.docx]

**Supplementary Figure 3.** Association between delta serum phosphate levels and 28-day mortality using a RCS curve. The solid line and shadow represented the HR of 28-day mortality and 95% CI, respectively. The RCS curve was adjusted for all covariates. RCS, restricted cubic spline; HR, hazard ratio; CI, confidence interval.
