# Supplementary material for: Association of serum phosphate and changes in serum phosphate with 28-day mortality in septic shock from MIMIC-IV database
Source: Sci Rep. 2023 Dec 10;13:21869. doi: 10.1038/s41598-023-49170-6 (PMC10711004; doi:10.1038/s41598-023-49170-6)
Supplement: Supplementary file 7 — Supplementary Information 7. [file 41598_2023_49170_MOESM7_ESM.docx]

**Supplementary Table 1.** Characteristics of patients based on 28-day mortality

|  | Total (n=3296) | Survivors (n=2307) | Non-survivors (n=989) | *P*-value |
| --- | --- | --- | --- | --- |
| Age (years) | 68.57 (57.1, 79.84) | 67.41 (55.72, 78.64) | 72.34 (60.63, 81.76) | < 0.001 |
| Gender, n (%) |  |  |  | 0.981 |
| Female | 1487 (45.12) | 1040 (45.08) | 447 (45.2) |  |
| Male | 1809 (54.88) | 1267 (54.92) | 542 (54.8) |  |
| Weight (kg) | 79.22 (65.78, 97.01) | 79.38 (66.39, 97.24) | 78.9 (64.8, 96) | 0.221 |
| Temperature (℃) | 36.91 (36.62, 37.31) | 36.96 (36.67, 37.37) | 36.78 (36.46, 37.15) | < 0.001 |
| MAP (mm Hg) | 74.26 (70.06, 79.31) | 74.73 (70.72, 79.67) | 73.03 (68.52, 78.32) | < 0.001 |
| SpO_2_ (%) | 96.8 (95.37, 98.18) | 96.93 (95.6, 98.23) | 96.44 (94.83, 98.08) | < 0.001 |
| Heart rate (bpm) | 92.23 (79.94, 105.03) | 91.19 (79.4, 103.81) | 94.98 (81.33, 107.23) | < 0.001 |
| Respiratory rate (bpm) | 21.06 (18.19, 24.25) | 20.72 (17.96, 23.92) | 21.85 (18.82, 25.26) | < 0.001 |
| WBC (10^9^/L) | 14.1 (8.8, 20.3) | 14.1 (9, 20.4) | 14.1 (8.3, 20.2) | 0.359 |
| Creatinine (mg/dL) | 1.4 (0.9, 2.4) | 1.3 (0.9, 2.2) | 1.6 (1.1, 2.7) | < 0.001 |
| Sodium (mmol/L) | 138 (134, 141) | 138 (135, 141) | 138 (134, 142) | 0.849 |
| Calcium (mg/dL) | 7.8 (7.3, 8.4) | 7.8 (7.3, 8.3) | 7.9 (7.2, 8.5) | 0.027 |
| Lactate (mmol/L) | 2.2 (1.5, 3.5) | 2 (1.4, 3.2) | 2.6 (1.7, 4.45) | < 0.001 |
| Infection site, n (%) |  |  |  |  |
| Respiratory system | 1268 (38.47) | 859 (37.23) | 409 (41.35) | 0.029 |
| Urinary system | 855 (25.94) | 644 (27.92) | 211 (21.33) | < 0.001 |
| Digestive system | 796 (24.15) | 594 (25.75) | 202 (20.42) | 0.001 |
| Other sites | 982 (29.79) | 661 (28.65) | 321 (32.46) | 0.032 |
| Congestive heart failure, n (%) | 1093 (33.16) | 734 (31.82) | 359 (36.3) | 0.014 |
| Chronic pulmonary disease, n (%) | 909 (27.58) | 614 (26.61) | 295 (29.83) | 0.064 |
| Diabetes, n (%) | 1052 (31.92) | 738 (31.99) | 314 (31.75) | 0.924 |
| Renal disease, n (%) | 820 (24.88) | 540 (23.41) | 280 (28.31) | 0.003 |
| Liver disease, n (%) | 811 (24.61) | 471 (20.42) | 340 (34.38) | < 0.001 |
| Malignant tumor, n (%) | 671 (20.36) | 421 (18.25) | 250 (25.28) | < 0.001 |
| Cerebrovascular disease, n (%) | 338 (10.25) | 224 (9.71) | 114 (11.53) | 0.130 |
| Peripheral vascular disease, n (%) | 351 (10.65) | 224 (9.71) | 127 (12.84) | 0.009 |
| RRT, n (%) | 671 (20.36) | 357 (15.47) | 314 (31.75) | < 0.001 |
| Ventilation, n (%) | 2140 (64.93) | 1356 (58.78) | 784 (79.27) | < 0.001 |
| Vasopressor use, n (%) | 2826 (85.74) | 1924 (83.4) | 902 (91.2) | < 0.001 |
| SAPS II | 47 (37, 58) | 44 (35, 53) | 55 (46, 65) | <0.001 |
| SOFA | 10 (7, 13) | 9 (7, 12) | 12 (9, 15) | <0.001 |
| Serum phosphate (mg/dL) | 3.7 (2.8, 4.9) | 3.5 (2.7, 4.6) | 4.2 (3.2, 5.5) | < 0.001 |

MAP, mean arterial pressure; SpO_2_, saturation of peripheral oxygen; WBC, white blood cell; RRT, renal replacement therapy; SAPS II, simplified acute physiology score II; SOFA, sequential organ failure assessment.

**Supplementary Table 2.** Comparison of blood phosphate levels between the non-survivors group and the survivors group grouped by RRT treatment.

|  | Non-survivors | | | Survivors | |
| --- | --- | --- | --- | --- | --- |
| RRT | Serum phosphate Mean | Serum phosphate  standard Deviation |  | Serum phosphate Mean | Serum phosphate  standard Deviation |
| No | 4.14 | 1.65 |  | 3.56 | 1.41 |
| Yes | 5.35 | 2.13 |  | 5.20 | 2.11 |

RRT, renal replacement therapy

**Supplementary Table 3.** Univariate and multivariate analysis for 28-day mortality

|  | Univariate analysis | | Multivariate analysis | |
| --- | --- | --- | --- | --- |
|  | HR (95% CI) | *P*-value | HR (95% CI) | *P*-value |
| Age (years) | 1.01 (1.01 - 1.02) | <0.001 | 1.07 (1.02 - 1.12) | 0.003 |
| Gender, n (%) | 1.00 (0.88 - 1.13) | 0.957 | 0.98 (0.86 - 1.12) | 0.802 |
| Weight (kg) | 1.00 (1.00 - 1.00) | 0.419 | 1.00 (1.00 - 1.00) | 0.689 |
| Temperature (℃) | 0.65 (0.60 - 0.71) | <0.001 | 0.77 (0.70 - 0.84) | <0.001 |
| MAP (mm Hg) | 0.98 (0.97 - 0.98) | <0.001 | 0.98 (0.97 - 0.99) | <0.001 |
| SpO_2_ (%) | 0.91 (0.89 - 0.92) | <0.001 | 0.96 (0.94 - 0.98) | <0.001 |
| Heart rate (bpm) | 1.01 (1.00 - 1.01) | <0.001 | 1.01 (1.01 - 1.01) | <0.001 |
| Respiratory rate (bpm) | 1.05 (1.04 - 1.06) | <0.001 | 1.02 (1.01 - 1.04) | <0.001 |
| WBC (10^9^/L) | 1.00 (0.99 - 1.00) | 0.429 | 1.00 (0.99 - 1.00) | 0.252 |
| Creatinine (mg/dL) | 1.07 (1.04 - 1.11) | <0.001 | 0.94 (0.89 - 1.00) | 0.036 |
| Sodium (mmol/L) | 1.00 (0.99 - 1.01) | 0.981 | 0.99 (0.99 - 1.01) | 0.163 |
| Calcium (mg/dL) | 1.07 (1.00 - 1.15) | 0.053 | 1.08 (1.01 - 1.16) | 0.027 |
| Lactate (mmol/L) | 1.15 (1.12 - 1.18) | <0.001 | 1.01 (0.98 - 1.04) | 0.409 |
| Infection site, n (%) |  |  |  |  |
| Respiratory system | 1.13 (0.99 - 1.28) | 0.066 | 0.84 (0.69 - 1.04) | 0.108 |
| Urinary system | 0.73 (0.63 - 0.85) | <0.001 | 0.73 (0.60 - 0.89) | 0.002 |
| Digestive system | 0.77 (0.66 - 0.90) | 0.001 | 0.67 (0.54 - 0.83) | <0.001 |
| Other sites | 1.20 (1.05 - 1.37) | 0.008 | 0.98 (0.76 - 1.26) | 0.876 |
| Congestive heart failure, n (%) | 1.16 (1.02 - 1.32) | 0.028 | 0.96 (0.83 - 1.10) | 0.536 |
| Chronic pulmonary disease, n (%) | 1.14 (0.99 - 1.30) | 0.068 | 1.06 (0.92 - 1.22) | 0.395 |
| Diabetes, n (%) | 0.98 (0.86 - 1.12) | 0.800 | 0.93 (0.81 - 1.08) | 0.341 |
| Renal disease, n (%) | 1.21 (1.05 - 1.39) | 0.007 | 1.10 (0.93 - 1.29) | 0.264 |
| Liver disease, n (%) | 1.77 (1.55 - 2.02) | <0.001 | 1.65 (1.42 - 1.92) | <0.001 |
| Malignant tumour, n (%) | 1.41 (1.22 - 1.63) | <0.001 | 1.34 (1.15 - 1.57) | <0.001 |
| Cerebrovascular disease, n (%) | 1.15 (0.95 - 1.40) | 0.158 | 1.19 (0.97 - 1.45) | 0.087 |
| Peripheral vascular disease, n (%) | 1.28 (1.06 - 1.54) | 0.009 | 1.06 (0.87 - 1.29) | 0.544 |
| RRT, n (%) | 2.05 (1.79 - 2.35) | <0.001 | 1.18 (1.00 - 1.41) | 0.056 |
| Ventilation, n (%) | 2.32 (1.99 - 2.70) | <0.001 | 1.65 (1.37 - 1.99) | <0.001 |
| Vasopressor use, n (%) | 1.92 (1.54 - 2.39) | <0.001 | 0.94 (0.74 - 1.20) | 0.615 |
| SAPS II | 1.04 (1.04 - 1.04) | <0.001 | 1.01 (1.00 - 1.02) | <0.001 |
| SOFA | 1.14 (1.12 - 1.16) | <0.001 | 1.06 (1.03 - 1.08) | <0.001 |
| Serum phosphate (mg/dL) | 1.20 (1.17 - 1.24) | <0.001 | 1.07 (1.02-1.12) | 0.003 |

MAP, mean arterial pressure; SpO_2_, saturation of peripheral oxygen; WBC, white blood cell; RRT, renal replacement therapy; SAPS II, simplified acute physiology score II; SOFA, sequential organ failure assessment; ICU, intensive care unit; HR, hazard ratio; CI, confidence interval.
